# Characterization of *Escherichia coli* Persisters from Biofilm Culture: Multiple Dormancy Levels and Multigenerational Memory in Formation

**DOI:** 10.3390/microorganisms12091888

**Published:** 2024-09-13

**Authors:** Hirona Ikeda, Sumio Maeda

**Affiliations:** Graduate School of Humanities and Sciences, Nara Women’s University, Kitauoya-nishimachi, Nara 630-8506, Japan

**Keywords:** *Escherichia coli*, modified cell filamentation method, dormancy, persister cells, viable but nonculturable cells, epigenetic memory

## Abstract

Persister cells (PCs), a subpopulation occurring within normal cells, exhibit a transient tolerance to antibiotics because of their dormant state. PCs are categorized into two types: type I PCs, which emerge during the stationary phase, and type II PCs, which emerge during the logarithmic phase. Using the conventional colony-forming method, we previously demonstrated that type I PCs of *Escherichia coli* form more frequently in air–solid biofilm culture than in liquid culture. In the current study, we modified a cell filamentation method as a more efficient and rapid alternative for quantifying PCs. This modified method yielded results consistent with those of the conventional method with 10^3^–10^4^ times higher sensitivity and less detection time, within several hours, and further revealed the existence of multiple levels of type I PCs, including a substantial number of deeply dormant cells. This study also discovered a potential epigenetic memory mechanism, spanning several generations (four or six cell divisions), which influences type II PC formation based on prior biofilm experience in *E. coli*.

## 1. Introduction

Bacterial cells spontaneously give rise to various subpopulations, including persister cells (PCs), as a survival strategy in response to environmental adaptations. PCs, which were first discovered in *Staphylococcus* sp. [1] and subsequently identified in other bacteria, including *Escherichia coli* [2,3,4], exhibit transient phenotypic tolerance to antibiotics due to their dormant state. This characteristic differentiates PCs from antibiotic-resistant cells, which exhibit permanent resistance due to genetic mutations or the acquisition of antibiotic resistance genes via horizontal gene transfer [5,6,7,8]. PCs are categorized into two types: type I PCs (PCs-I), which emerge during the stationary phase, and type II PCs (PCs-II), which emerge during the logarithmic phase [9,10]. Obtaining a comprehensive understanding of the nature and mechanisms of PCs is crucial for bacterial survival in antibiotic-positive human-related environments, including medical states [3,11]. However, despite extensive research, the detailed properties and mechanisms of PC formation remain elusive.

Viable but nonculturable (VBNC) cells share dormancy characteristics with PCs. Both PCs and VBNC cells exhibit antibiotic tolerance due to dormancy and have been detected as viable cells via live cell staining [12,13]. However, they differ in their resuscitation abilities: PCs are easily resuscitated, whereas VBNC cells are not [13]. Recent studies have proposed that PCs and VBNC cells exist on a dormancy continuum, in which PCs are lightly dormant and easily reverting to normal proliferative cells, whereas VBNC cells are deeply dormant and more challenging to activate [12,14,15]. Consistent with this concept, recent studies have shown that VBNC cells can revert to colony-forming cells under certain conditions, such as varying nutrient conditions or the application of activating stimuli [16,17,18,19,20]. Based on this knowledge, the current study equates VBNC cells with PCs.

Recently, we reported that *E. coli* PCs-I form more frequently in air–solid biofilm culture (BC) than in liquid culture (LC). Moreover, we revealed that these BC-derived PCs-I can maintain their PC phenotype for up to 4 weeks in a fresh antibiotic-containing medium even after removal from BC. This finding suggests the existence of a long retention effect or nonepigenetic memory effect in the planktonic state of *E. coli* cells [21]. In general, transient memory phenomena of cellular experiences are classified into two categories: epigenetic and nonepigenetic. The distinction between these two categories lies in the presence or absence of the effect across cell generations, respectively. To date, evidence regarding bacterial memory effects, whether epigenetic [18,22,23,24,25] or nonepigenetic [21,26], has been gradually accumulating in various bacterial phenomena.

In our previous study [21], we quantified PCs-I using the conventional colony-forming method (hereafter abbreviated as “colony method”). However, although this method is commonly used for PC detection and counting, it is time-consuming, labor-intensive, and costly, especially when handling multiple samples. Additionally, it requires the resuscitation of dormant PCs into proliferating cells for detection, potentially leading to an underestimation of the total PCs, including VBNC cells. As the first aim of this study, to address these issues, we attempted to establish an alternative method by combining the cephalexin (Cep)-induced cell filamentation method developed by Windels et al. [27] with common cell viability assays using propidium iodide (PI) or cyanoditolyl tetrazolium chloride (CTC) under a fluorescence microscope. Windels et al. [27] reported that for PC detection, the advantage of using Cep, which targets penicillin-binding protein 3, essential for septum synthesis, is its characteristic of inducing extensive filamentation of non-PCs before the initiation of lysis, enabling us to clearly distinguish PCs and non-PCs. This cell filamentation method has also been used in other studies; for example, it was used in combination with flow cytometry in order to characterize and quantify PCs at the single-cell level [13,28]. We applied the new method, termed as the “modified cell filamentation method” (hereafter abbreviated as ”filamentation method”), to reanalyze the previously reported enhanced formation of PCs-I via BC in *E. coli*. Our results confirmed that the filamentation method is effective in detecting PCs-I, showing a higher incidence in BC-derived cells than in LC-derived cells. Moreover, we revealed a greater number of PCs using the filamentation method than using the colony method, indicating the ability of the filamentation method to detect a large number of cells in deep dormancy, in addition to those in light dormancy that were detectable via the colony method. Finally, we used the filamentation method to analyze the formation of PCs-II in LC- and BC-derived *E. coli* cells, revealing for the first time the involvement of a possible epigenetic memory mechanism spanning multiple generations in PC-II formation directed by prior biofilm experience.

## 2. Materials and Methods

### 2.1. Bacterial Strains and Materials

The *E. coli* strains used in this study, namely MG1655 (F−, λ−, *rph-1*) [29], W3110 [F−, λ−, *IN(rrnD-rrnE)1, rph-1*] [29], and BW25113 [F−, r*rnB, ΔlacZ4787, HsdR514, Δ(araBAD)567, Δ(rhaBAD)568, rph-1*] [30], were sourced from the NBRP (NIG, Mishima, Japan). Luria–Bertani (LB) powder (Lennox) was procured from Sigma (St. Louis, MO, USA). Dulbecco’s phosphate-buffered saline (PBS) tablets (without calcium and magnesium) were obtained from KAC (Hyogo, Japan). Biodyne A nylon membrane filters (pore size: 0.45 µm) were purchased from Pall (Port Washington, NY, USA). PI solution and a CTC Rapid Staining Kit for Microscopy were acquired from DOJINDO (Kumamoto, Japan). Syringe filters (pore size: 0.20 µm) were sourced from Iwaki (Tokyo, Japan). Methyl pyruvate ester was procured from Tokyo Chemical Industry (Tokyo, Japan). Cep, sodium pyruvate, glycerol, D(+)-glucose, sterile water, agar powder (guaranteed-reagent grade), and other general reagents were obtained from FUJIFILM Wako Pure Chemical Co. (Osaka, Japan).

### 2.2. BC and LC of E. coli before PC Measurement

For all experiments described below, the cell numbers set in each experiment were values that were determined in advance based on the results of past research [21,26] and trial experiments to ensure that good results could be obtained in terms of reproducibility and quantitativeness in the current experiments.

BC and LC of *E. coli* cells were conducted as previously described [21,31]. Initially, cells were precultured in LB broth at 37 °C for 16 h under shaking conditions at 150 rpm. Following this, 1 mL of the culture solution was centrifuged and resuspended in 1 mL of fresh PBS. The turbidity (OD_600_) of an aliquot of the solution was measured to estimate the total cell number (OD_600_ of 1.0 = 6.0 × 10^8^ cells/mL). Cells were then recovered via centrifugation, and the cell density was adjusted to 9 × 10^8^ cells/mL with fresh LB broth. For LC, aliquots (15 µL; total 1.35 × 10^7^ cells) of the prepared cell suspension were inoculated into test tubes containing 3 mL of fresh LB broth and cultured at 37 °C for 20 h under shaking conditions at 150 rpm. For BC, equivalent aliquots of the prepared cells were inoculated onto LB agar plates covered with sterilized nylon membrane filters and cultured at 37 °C for 20 h.

### 2.3. Incidence Measurement of Cep-Tolerant PCs-I Using the Colony Method

BC cells were retrieved and suspended in 1.8 mL of fresh LB broth. Subsequently, 1 mL of LC or BC suspension was centrifuged and then resuspended in 1 mL of fresh PBS. Turbidity (OD_600_) measurements for aliquots of samples were obtained to estimate the total cell number for each sample. A portion of each culture solution was spread onto LB agar to determine the initial number of colony-forming units (CFUs). Sample aliquots, each containing 5.2 × 10^6^ cells, were transferred to 15 mL centrifuge tubes and suspended in 4 mL of fresh LB broth containing 400 µg/mL Cep. The centrifuge tubes were then incubated at 37 °C for 6 h with their lids securely closed while being shaken at 150 rpm. After the incubation period, the Cep-treated cells were collected via centrifugation, washed once with fresh PBS, and then resuspended in 200 µL of fresh PBS. Subsequently, the suspensions were serially diluted and spread onto antibiotic-free LB agar plates, which were then incubated at 37 °C overnight to measure the final number of CFUs in each sample. The incidences of PCs were calculated as the survival rate of cells during the incubation period in the Cep-containing media, specifically as the ratio of the final CFU count to the initial CFU count.

### 2.4. Incidence Measurement of PCs-I Using the Filamentation Method

For the filamentation method based on the method by Windels et al. [27], we conducted preliminary experiments to optimize the conditions for Cep treatment and PC detection. The protocols for cell culture and Cep treatment were essentially identical to those described in the previous section, with the exception that Cep was applied at a concentration of 100 µg/mL for 2 h. Following Cep treatment, the resulting cell/PBS suspensions (200 µL) were transferred to 1.5 mL microtubes. The protocols used for PI and CTC staining were based on the manufacturer’s instructions (https://www.dojindo.com/manual/BS02/) (accessed on 6 September 2024). For PI staining, we added 0.2 µL of PI stock solution (1 mg/mL) to the cell/PBS suspension, which was then incubated at room temperature for 5 min. For CTC staining, we added 6 µL of CTC stock solution (15.6 mg/mL) and 1.5 µL of enhancing reagent B to the cell/PBS suspension. This mixture was then incubated at 37 °C for 30 min under shaking conditions at 350 rpm using a microplate shaker. The cell suspensions were subsequently centrifuged and resuspended in 20 µL of fresh PBS. These suspensions were used as specimens for phase-contrast microscopy and fluorescence microscopy, with G excitation for PI and B excitation for CTC. We calculated the incidence of PCs as the ratio of nonfilamentous living cells to the total number of living cells.

### 2.5. Resuscitation of Deeply Dormant PCs-I Using Various Additives

Cell culture, Cep treatment, and cell plating were performed in the same manner as described in Section 2.3, except that the cells were plated onto agar medium supplemented with each of the following additives: 4 mM sodium pyruvate, 16 mM methyl pyruvate, 0.5% (*v*/*v*) glycerol, and 0.1% (*w*/*v*) glucose [16,17,18,19,20].

### 2.6. Cell Culture for PC-II Formation and Measurement of PC-II Incidence Using the Filamentation Method

Following BC and LC at 37 °C for 20 h, the cells were collected via centrifugation and resuspended in fresh PBS. We then cultured aliquots of the cells (1.3 × 10^6^ cells/mL; approximately 1%–2% of the total cells in each sample) in 4 mL of fresh LB broth at 37 °C until they reached the logarithmic phase when PCs-II emerged. The number of cell divisions was estimated based on the culture time, which was correlated with the OD_600_ values obtained from previous trial experiments. Specifically, we estimated four cell divisions at 160 and 150 min for LC- and BC-derived cells, respectively, and six cell divisions at 245 and 220 min for LC- and BC-derived cells, respectively. After the logarithmic culture, the cells were collected, resuspended in fresh PBS, and then subjected to Cep treatment and PC detection using the filamentation method, similar to the process for PCs-I. We also calculated the incidence of PCs-II in the same manner as for PCs-I. Notably, to detect pure PCs-II in this experiment, measurement of PCs-II was performed until six cell divisions, because cell growth gradually slowed down soon after this time point, indicating that the cell population would enter a transition period to reach the stationary phase, in which new PCs-I were generated. Instead, to determine the fate of the generated PCs-II, the cells were successively cultured for 24 h until a stable stationary phase was reached, and total PCs at this endpoint were measured using the same filamentation method.

In addition, we considered the theoretical possibility of carryover of PCs-I into the PC-II count in this experiment. However, even if PCs-I were carried over through several steps in this experiment, their maximum contributions to the total PCs, based on our experimental data, were calculated to be <3.6% at four cell divisions and <1.0% at six cell divisions in any sample. We considered these small values as negligible and adopted the data of total PCs as those of PCs-II in this experiment. In fact, through trial calculations with subtraction of the estimated number of residual PCs-I, we confirmed that such a trace number of PCs-I, if present, did not influence the interpretation of the data of PCs-II.

### 2.7. Confirmation of No Involvement of Genetic Changes in PC-II Formation and Reversibility of PCs-II to Normal Cells

Following the first PC-II selection from BC- or LC-derived cells using the colony method, some of the resulting colonies were used for subsequent BC and LC. A portion of these cultured cells were then recultured until they reached the logarithmic phase, at which time point the second PC-II selection was performed using the filamentation method. The PCs-II were formed under conditions that allowed the cell population to divide four times. The protocols followed in each step of this experiment were identical to those used in corresponding experiments described previously.

### 2.8. Confirmation of No Involvement of Extracellular Materials Derived from BC or LC in PC-II Formation

The solution containing the extracellular materials of LC was the culture supernatant of LC itself. The cells were completely removed from this solution via centrifugation and filtration using a syringe membrane filter with a pore size of 0.20 µm. For BC, the solution containing extracellular materials was prepared differently. After BC, the cells were recovered with 1.8 mL of LB broth. This suspension was then centrifuged, and the supernatant was filtered in the same manner as that described for LC supernatant. Overall, 4 µL of each solution was added to 4 mL of fresh LB broth. This mixture was used for the logarithmic culture of another set of BC- or LC-derived cells and subsequent PC-II selection using the filamentation method. The PCs-II were formed under conditions that allowed the cell population to divide four times. The protocols followed in each step from BC or LC to PC-II selection were identical to those used in corresponding experiments described previously.

### 2.9. Statistical Analysis

For statistical analysis, we primarily used the *t*-test. However, for comparing multiple groups, we first performed a one-way analysis of variance (ANOVA), followed by a *t*-test for pairwise comparisons.

## 3. Results

### 3.1. The Filamentation Method Is a Useful Alternative to the Colony Method for Detecting PCs as Antibiotic-Tolerant Dormant Cells

**Figure 1:**
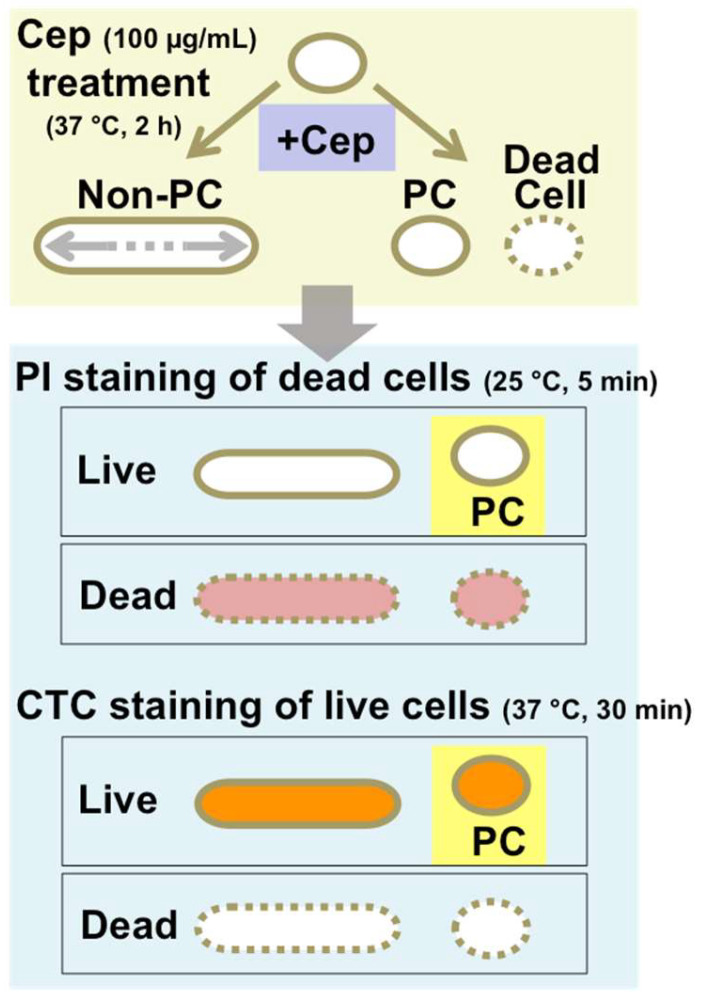
Schema of the experimental flow of the modified filamentation method. Cep treatment of the cell population produces filamentous non-PCs in addition to unaltered PCs and dead cells. Subsequent cell staining with PI or CTC can detect live and dead cells, enabling the identification of PCs as PI-negative nonfilamentous cells or CTC-positive nonfilamentous cells. Details of the experimental conditions are described in Materials and Methods Section 2.4.

To develop a new method for PC detection as an alternative to the colony method, we decided to modify a previously reported cell filamentation method by Windels et al. [27]. This method operates on the principle that Cep induces filamentation in normal cells, with the exception of dormant PCs or dead cells, under cell growth conditions. In the original method, filtration is used to isolate a nonfilamentous PC fraction following Cep treatment. However, for rapid PC detection and quantification in our modified filamentation methods, we replaced filtration with cell viability assays using PI or CTC, conducted under a fluorescence microscope immediately after Cep treatment (Figure 1).

**Figure 2:**
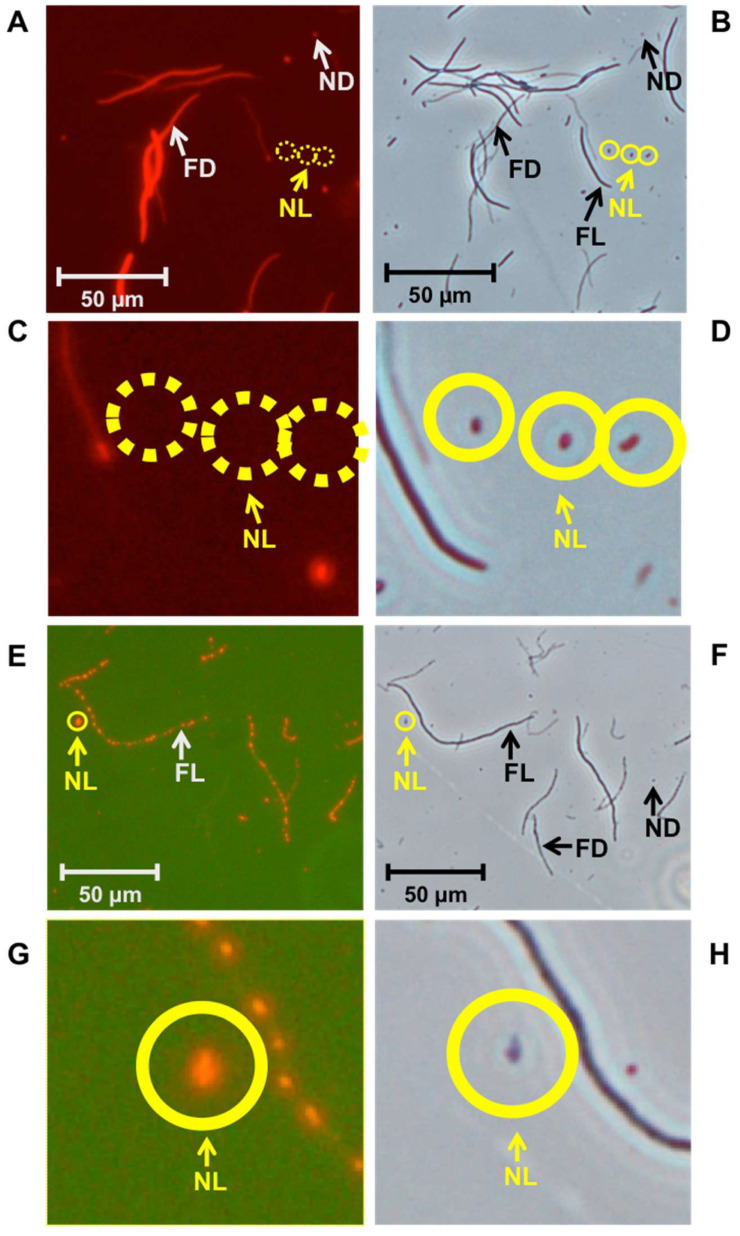
Micrographs of cells stained with PI (**A**,**C**) or CTC (**E**,**G**), as observed under a fluorescence microscope. The corresponding phase-contrast microscopy images are shown in (**B**,**D**,**F**,**H**). These images were obtained after applying the filamentation method to detect the PCs-I of BC-derived MG1655 cells. The filamentation method identified four types of cells: filamentous living cells (FL), filamentous dead cells (FD), nonfilamentous living cells (NL, representing PCs-I, which are circled in yellow, with solid lines indicating visible cells and dotted lines indicating nonvisible cells), and nonfilamentous dead cells (ND). (**C**,**D**,**G**,**H**) are magnified images focusing on “NL” in panels (**A**,**B**,**E**,**F**), respectively.

As demonstrated in Figure 2, the filamentation method, when used with PI or CTC for an *E. coli* MG1655 strain, successfully detected four types of cells: filamentous living cells, filamentous dead cells, the target nonfilamentous living cells (corresponding to PCs), and nonfilamentous dead cells. This confirms that the filamentation method can be effectively used for detecting dormant living PCs.

### 3.2. The Filamentation Method Can Substitute for the Colony Method

**Figure 3:**
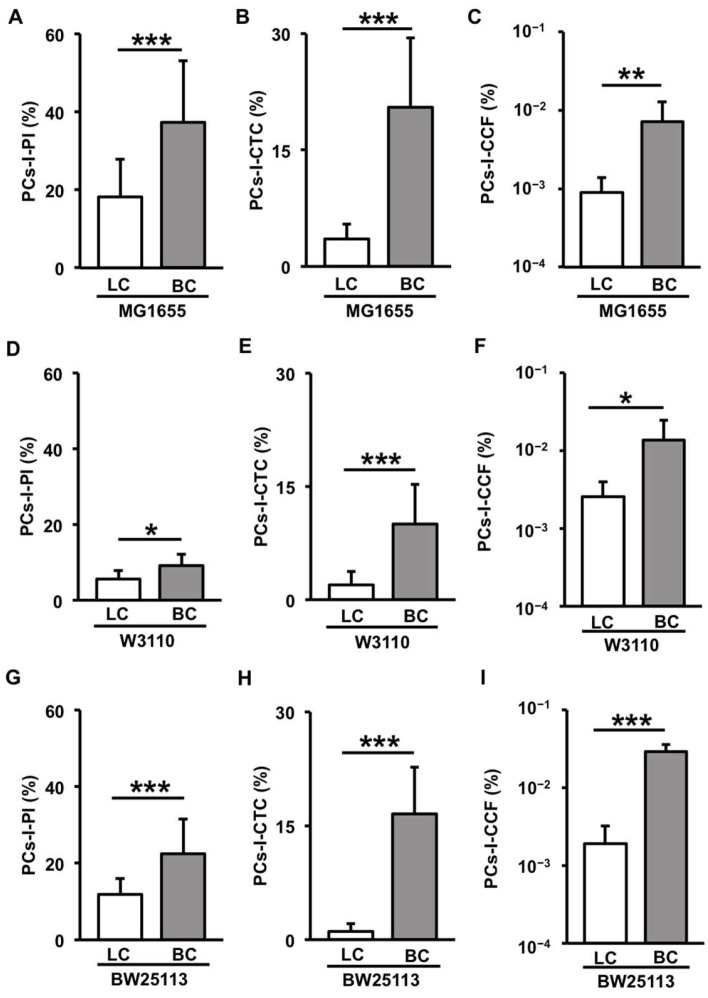
Comparison of PCs-I between cells derived from LC (white bars) and BC (gray bars) across three *E. coli* strains [MG1655 (**A**–**C**), W3110 (**D**–**F**), and BW25113 (**G**–**I**)]. The comparison was performed using three detection protocols: filamentation method-PI (**A**,**D**,**G**), filamentation method-CTC (**B**,**E**,**H**), and colony method (**C**,**F**,**I**). The PC-I incidence (%) data are presented as means and standard deviations (S.D.). Statistical significance was determined using a *t*-test (* *p* < 0.05; ** *p* < 0.01; *** *p* < 0.005, n = 7–30).

In our previous research using the colony method, we observed that antibiotic-tolerant PCs-I appeared more frequently in BC-derived cells than in LC-derived cells [21]. In this study, we aimed to confirm whether a high incidence of PCs-I in BC-derived cells could be replicated using the filamentation method. Figure 3A–C presents the quantification results of PI-negative PCs-I (Figure 3A), CTC-positive PCs-I (Figure 3B), and colony-forming PCs-I (Figure 3C) in an experiment involving the MG1655 strain subjected to Cep treatment following BC or LC. The colony-forming PCs-I, PI-negative PCs-I, and CTC-positive PCs-I showed a similar trend to that reported in a previous study using Amp treatment [21], i.e., the incidence of PCs-I was higher in BC-derived cells than in LC-derived cells. We obtained similar results with other *E. coli* strains, W3110 and BW25113 (Figure 3D–I). These findings validated the effectiveness of the filamentation method as a convenient alternative to the colony method for quantifying PCs.

### 3.3. The Filamentation Method Can Detect a Higher Number of PCs than the Colony Method

**Figure 4:**
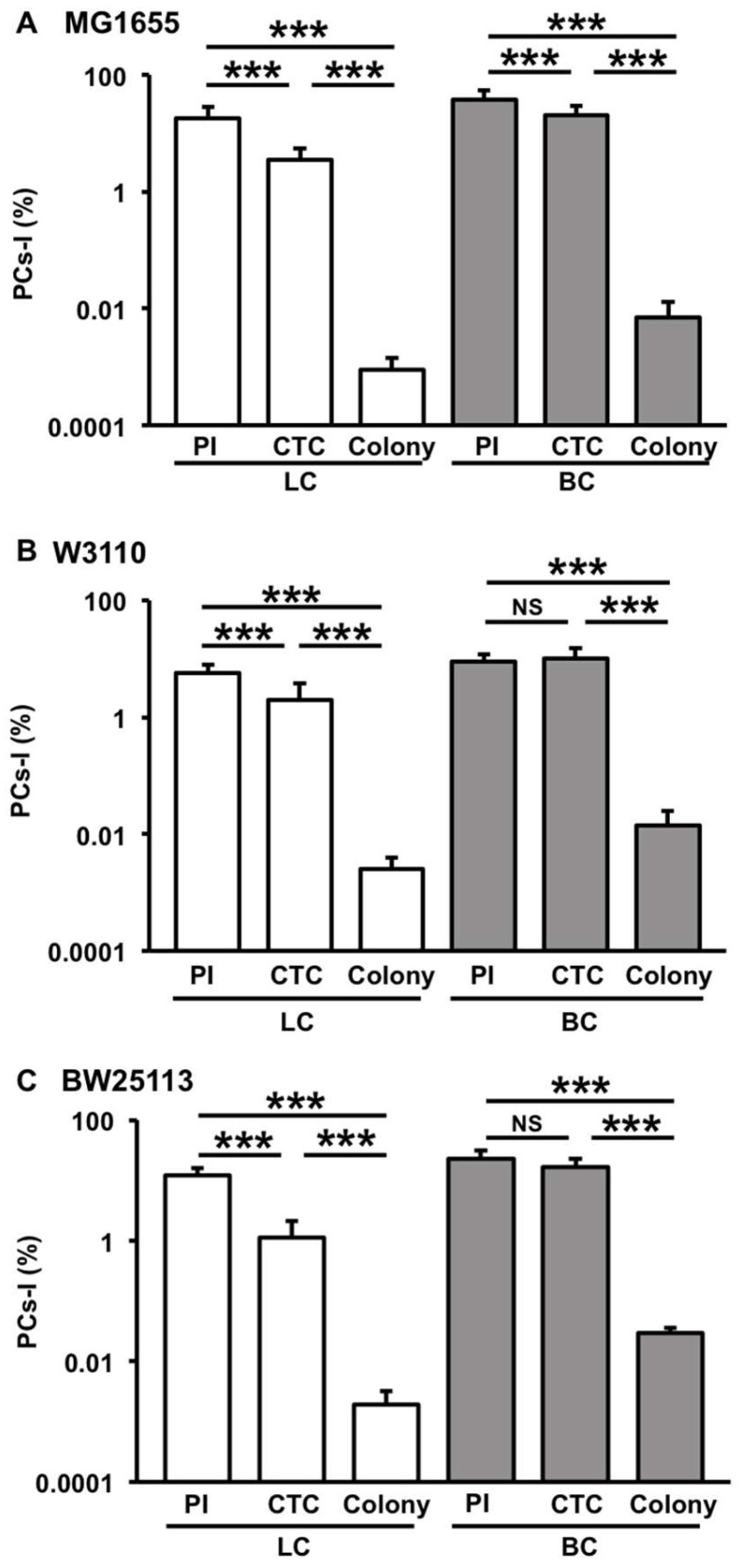
Comparison of PCs-I between the three detection protocols (PI: filamentation method-PI, CTC: filamentation method-CTC, and Colony: colony method) in cells derived from LC (white bars) and BC (gray bars) across three *E. coli* strains ((**A**) MG1655, (**B**) W3110, and (**C**) BW25113). The PC-I incidence (%) data are presented as means and S.D. Statistical significance was determined using one-way ANOVA followed by a *t*-test between each group (*** *p* < 0.005, n = 7–30, NS: not significant).

We compared the PC incidence among the three protocols on the MG1655 strain (Figure 4A). The PC detection rate of both the filamentation method with PI and the filamentation method with CTC was 10^3^–10^4^ times higher than that of the colony method in both BC- and LC-derived cells. The number of PI-negative PCs-I was approximately 3–5 times greater than that of CTC-positive PCs-I. However, the difference between these two values was considerably smaller than the difference of either value and the number of colony-forming PCs-I. We observed similar trends with other *E. coli* strains, W3110 and BW25113 (Figure 4B,C). These results suggest the presence of multiple dormant levels of PCs-I and indicate that the filamentation method is capable of detecting a significantly larger number of PCs than the colony method.

### 3.4. PCs Detected Using the Filamentation Method Contain Cells That Are Deeply Dormant but Possess Potential to Reproliferate

**Figure 5:**
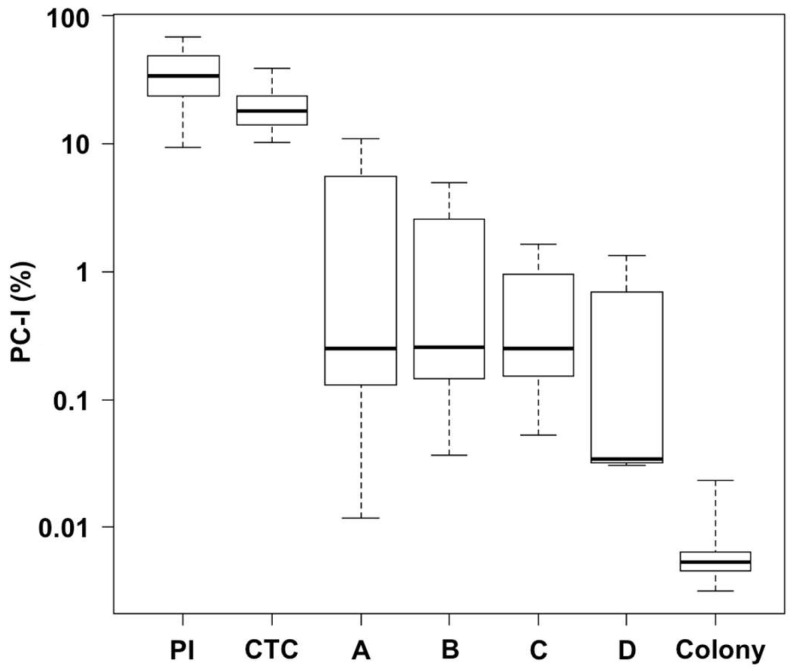
Resuscitation of deeply dormant PCs-I derived from BC of MG1655 by supplementation of nutrients [A: 4 mM sodium pyruvate, B: 16 mM methyl pyruvate, C: 0.5% (*v*/*v*) glycerol, and D: 0.1% (*w*/*v*) glucose]. The results are compared with controls [colony-forming PCs-I (Colony), PI-negative PCs-I (PI), and CTC-positive PCs-I (CTC)]. The data are presented as a box-and-whisker plot.

**Figure 6:**
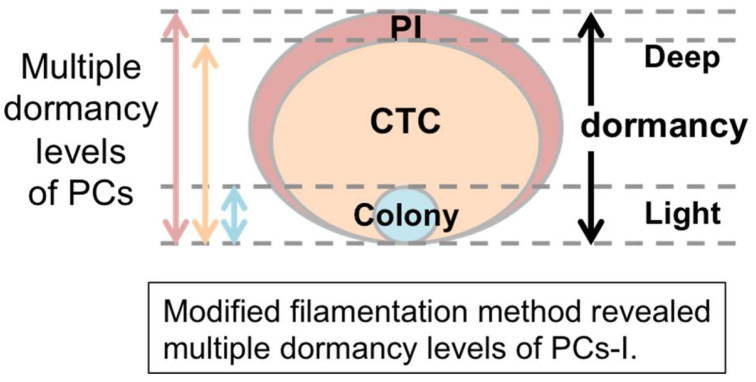
Schema of multiple dormancy levels of PCs-I revealed via the filamentation method. This illustrates a probable relationship of three groups of PCs [PI-negative PCs-I (PI), CTC-positive PCs-I (CTC), and colony-forming PCs-I (Colony)], all of which exist on a gradually dormant continuum.

As shown in Figure 4, the PI-negative PC-I and CTC-positive PC-I samples seem to contain a considerable proportion of VBNC cells that are challenging to resuscitate. Recent studies have shown that VBNC cells can revert to a colony-forming state under certain culture conditions, such as varying nutrient conditions [16,17,18,19,20]. However, these studies are ongoing. Our experiments aimed to show that the PI-negative PC-I and CTC-positive PC-I samples contain cells that have the potential to proliferate under specific culture conditions. After Cep treatment, we supplied the cells with four nutrients (sodium pyruvate, methyl pyruvate, glycerol, and glucose) that are known to possess some resuscitation activity on VBNC cells. Figure 5 presents the results of this experiment using BC-derived cells. Post-Cep treatment, these cells were plated onto agar medium supplemented with additives. Despite substantial data fluctuations (as evidenced by the box-and-whisker plot in Figure 5), all additives tended to yield more colonies than the control group without additives. In particular, the maximum value of the sodium pyruvate data (plot A) reached the incidence range of PI-negative PCs-I and CTC-positive PCs-I. We observed similar effects of these additives in an experiment using LC-derived cells (Figure S9). These results suggest that our PI-negative PC-I and CTC-positive PC-I samples contain a substantial number of cells that can proliferate under suitable culture conditions. This finding aligns with the recently proposed concept that PCs and VBNC cells exist on a continuum of dormancy [12,14,15]. Therefore, the PI-negative PC-I and CTC-positive PC-I fractions can be categorized as PCs. Notably, based on the data from Figure 4 and Figure 5, the colony method likely underestimates the PC incidence by 3–4 orders of magnitude compared with the filamentation method. Figure 6 illustrates the model of multiple dormancy levels of PCs-I deduced from our results.

### 3.5. The Filamentation Method Is Also Effective in Detecting PCs-II

**Figure 7:**
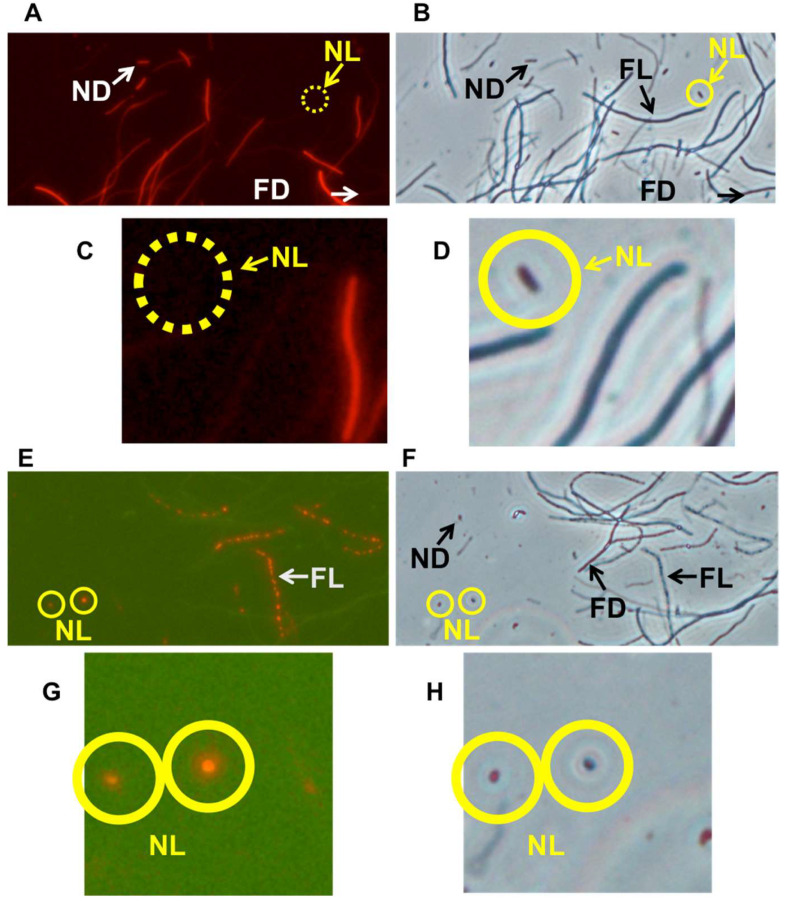
Micrographs of cells stained with PI (**A**,**C**) or CTC (**E**,**F**), as observed under a fluorescence microscope. The corresponding phase-contrast microscopy images are shown in (**B**,**D**,**F**,**H**). These images were obtained after applying the filamentation method to detect the PCs-II of BC-derived MG1655 cells. The filamentation method identified four types of cells: filamentous living cells (FL), filamentous dead cells (FD), nonfilamentous living cells (NL, representing PCs-II, which are circled in yellow, with solid lines indicating visible cells and dotted lines indicating nonvisible cells), and nonfilamentous dead cells (ND). (**C**,**D**,**G**,**H**) are magnified images focusing on “NL” in panels (**A**,**B**,**E**,**F**), respectively.

We utilized the filamentation method to detect PCs-II produced during the logarithmic culture phase. Microscopic observations confirmed the presence of nonfilamentous living cells that corresponded to PCs-II, along with three other types of cells (Figure 7). The living cells were equivalent to PCs-I, indicating that the filamentation method is also effective in detecting PCs-II.

### 3.6. BC-Derived Cells Produce PCs-II More Frequently than LC-Derived Cells for Multigenerations after Their Culture

**Figure 8:**
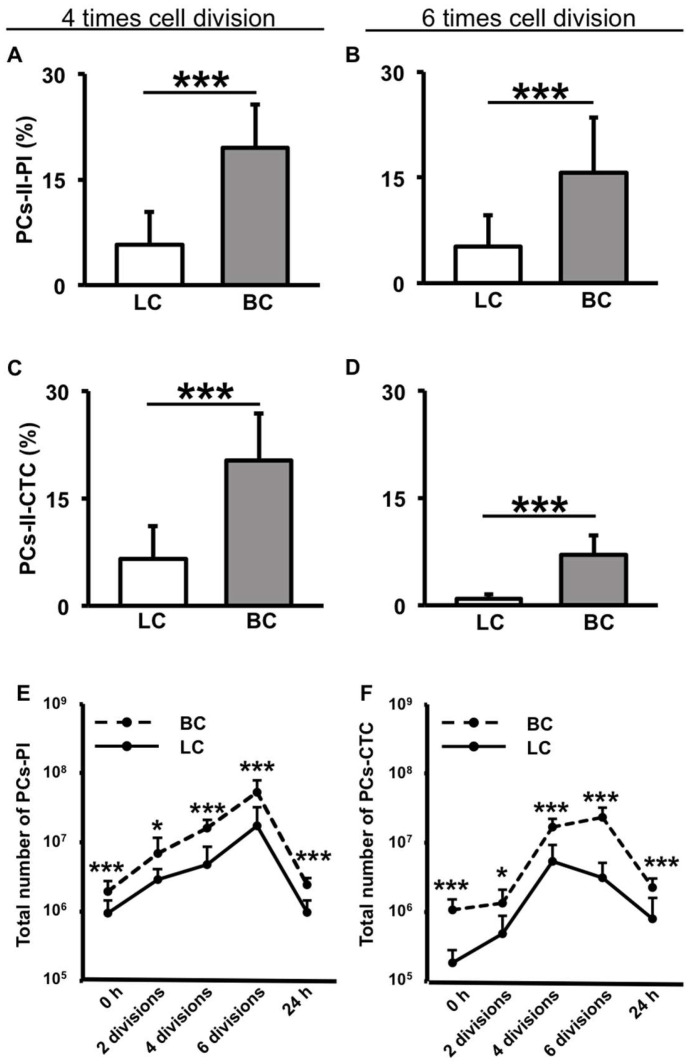
Comparison of PCs-II between cells derived from LC (white bars) and BC (gray bars) of MG1655 after 4 or 6 cell divisions (left panels (**A**,**C**) and right panels (**B**,**D**), respectively). The comparison was performed using the filamentation method-PI (**A**,**B**) and the filamentation method-CTC (**C,D**). Time courses of absolute numbers of PI-negative PCs (**E**) or CTC-positive PCs (**F**) per culture of BC- and LC-derived cells (0 h—culture start point, 2–6 divisions—number of cell divisions in the log phase, and 24 h—culture endpoint reaching the stable stationary phase). The PC incidence (%) data are presented as means and standard deviations (S.D.). Statistical significance was determined using a *t*-test (* *p* < 0.05; *** *p* < 0.005, n = 8–15).

We examined the potential of BC- and LC-derived cells, which had reached the stationary phase, to produce PCs-II in a newly initiated logarithmic culture. Each aliquot was recultured under uniform conditions in fresh liquid media until the mid-logarithmic phase. We then measured the incidence of PCs-II using the filamentation method. As shown in Figure 8A–D, the results after four or six cell divisions consistently showed that PCs-II were produced more frequently in BC-derived cells than in LC-derived cells. This suggests that, similar to PCs-I, PCs-II are preferably produced in BC-derived cells. Interestingly, these results indicate that the influence of the initial BC and LC on the subsequent incidence of PCs-II persists across multiple generations of cell division.

To demonstrate the generation, increase, and fate of PCs-II during this culture, the time courses of the absolute numbers of PI-negative PCs or CTC-positive PCs per culture are presented in Figure 8E,F. In these graphs, initial PCs are PCs-I carried over from the seed cells of BC- and LC-derived cells. With the progression of logarithmic growth (2, 4, and 6 cell divisions), the number of PCs gradually increased, demonstrating the generation and increase of PCs-II during this period. After continued culture up to 24 h to reach the stable stationary phase, the number of PCs largely decreased to the initial level of PCs, confirming the expected transient existence of PCs-II specific to the logarithmic phase. Even at this endpoint, more PCs were detected in BC-derived cells than in LC-derived cells; however, it remains unknown whether these PCs consisted of newly formed PCs-I alone or PCs-I with residual PCs-II.

### 3.7. Absence of Specific Genetic Changes and Extracellular Factors That Influence PC-II Incidence

**Figure 9:**
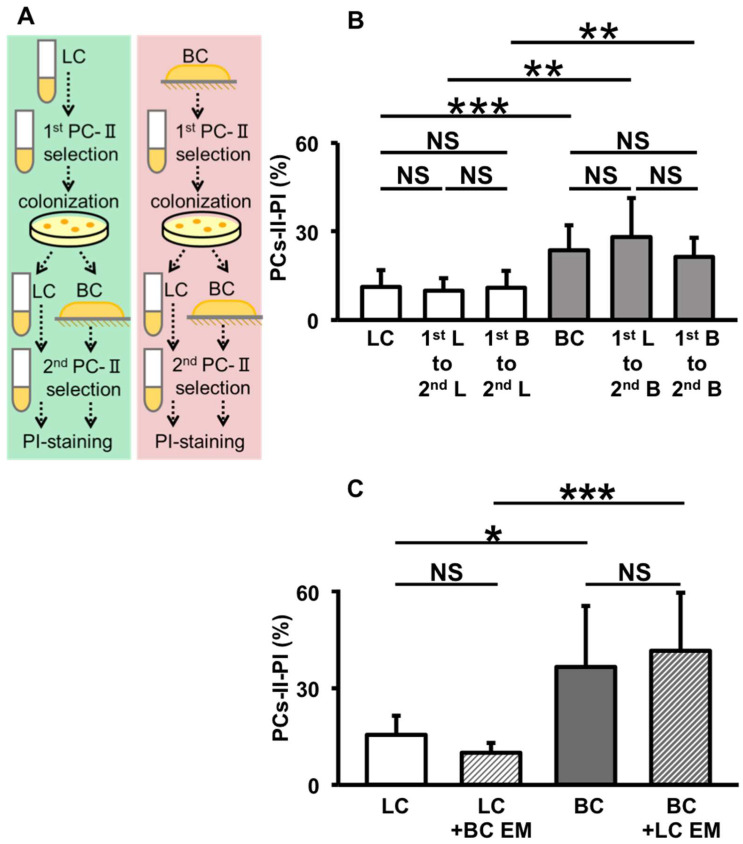
Effect of the first PC-II selection on the second PC-II formation ((**A**) schema of the experimental flow; (**B**) data graph) and the effect of extracellular materials from BC or LC on PC-II formation (**C**). In panel B, labels below each bar denote the experimental manipulations in each data set. For example, “LC” denotes LC followed by PC-II selection and detection via the filamentation method-PI; “1st B to 2nd L” denotes the first BC followed by the first PC-II selection and colonization and then the second LC followed by the second PC-II selection and detection using the filamentation method-PI. The other samples are denoted in the same manner. In panel C, labels below each bar denote the experimental manipulations in each data set. “LC” and “BC” denote PC-II formation in LC- and BC-derived cells with no supply, respectively. “LC + BC EM” denotes PC-II formation in LC-derived cells supplied with extracellular materials of BC, and “BC + LC EM” denotes PC-II formation in BC-derived cells supplied with extracellular materials of LC. In panels B and C, data regarding the PC-II incidence (%) are presented as means and S.D. Statistical significance was determined using one-way ANOVA followed by a *t*-test between each group (* *p* < 0.05; ** *p* < 0.01; *** *p* < 0.005, n = 7–9, NS: not significant).

We aimed to further determine whether the PCs-II detected in the experiment described in Figure 8 underwent any genetic changes that influenced the PC-II incidence. We also examined the reversibility of these PCs-II to normal cells and their behavior in subsequent PC-II formation. The protocol schema for this experiment is shown in Figure 9A. The first PCs-II were formed from BC- and LC-derived cells using the same protocol as in Figure 8. We then selected and colonized Cep-tolerant colony-forming PCs-II. Some of these colonies were used again for BC and LC and then for the second PC-II selection using the filamentation method. As shown in Figure 9B, the first PC-II selection did not influence the PC-II incidence in the second selection. Moreover, BC-derived cells produced PCs-II more frequently than LC-derived cells; this characteristic remained consistent across all samples. These results suggest that the PCs-II selected in the initial experiments could revert to unbiased normal cells and did not acquire new genetic changes that influenced the PC-II incidence.

In the Figure 8 experiment, we theorized that some extracellular factors that might influence the PC-II incidence were carried over from the prior BC or LC. However, their potential contribution, even if they existed, was considered minimal because the preparation steps of the inoculation cells removed almost all such materials. To experimentally rule out this possibility, we conducted an experiment for supplying extracellular materials of the BC or LC to the PC-II formation culture (Figure 9C). The results showed that supplying a solution containing such materials did not change the PC-II incidence in either BC- or LC-derived cells, even when BC- or LC-derived materials were added reciprocally to LC- or BC-derived cells, respectively. This suggests that extracellular factors carried over from the prior BC or LC do not influence later PC-II formation.

**Figure 10:**
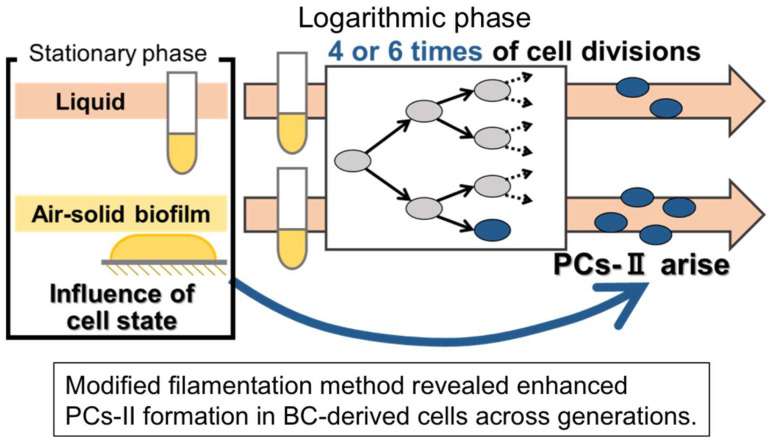
Conceptual diagram of the potential presence of an epigenetic memory mechanism in PC-II formation. This illustrates how the influence of the prior BC and LC in the stationary phase on the later PC-II formation in the logarithmic phase appears to extend beyond multiple generations of cell division.

Considering the results shown in Figure 8 and Figure 9 together, we interpreted that the former result does not stem from the influence of certain genetic changes or residual extracellular factors. Instead, it appears to result from a type of intracellular memory phenomenon wherein past culture experience is inscribed within the cells and carried across multiple generations (Figure 10). This suggests the presence of an epigenetic memory mechanism in PC-II formation.

## 4. Discussion

In this study, we enhanced the filamentation method for improved detection of *E. coli* PCs. This method allowed us to confirm the consistency in the increased occurrence of PCs-I in BC-derived cells between the filamentation method and the colony method. We also discovered multiple dormancy levels of PCs-I (as illustrated in Figure 6) and found that PC-II formation was more frequent in BC-derived cells than in LC-derived cells. This suggests that the influence of the initial BC and LC on the subsequent PC-II incidence persists across multiple generations of cell division, indicating the presence of a multigenerational memory mechanism in PC-II formation (as illustrated in Figure 10).

Our improved filamentation method can replace the colony method as a more rapid and sensitive approach for PC quantification. Compared with the original method by Windels et al. [27] that aimed to isolate PCs via filtration, our modified protocol was designed solely for PC quantification. In addition, the original method focused on PCs-II alone, whereas our method was demonstrated to detect both PCs-I and PCs-II.

The presence of multiple dormancy levels of PCs aligns with the recent concept proposed by Ayrapetyan et al. [12] that PCs and VBNC cells exist on a gradually dormant continuum. We confirmed that at least some subpopulations detectable using the filamentation method but not using the colony method can be resuscitated to generate colony-forming cells. Although full resuscitation of the dormant cells detected using the filamentation method was not achieved, the maximum resuscitation data in Figure 5 was within the data range of the filamentation method. This suggests that the dormant cells detected using the filamentation method have some potential for resuscitation. Notably, the simple colony method, without any resuscitation treatments, tended to significantly underestimate the PC incidence (Figure 5). The large fluctuation of the resuscitation data in Figure 5 is not unique to our experiment but has been commonly observed in similar studies [17,19,20]. Further research is warranted to address this issue of an unstable resuscitation efficiency for improving the colony method.

Furthermore, this study revealed two key findings: the more frequent production of PCs-II in BC-derived cells and the influence of prior culture on subsequent generations. The former indicates a new effect of biofilm on PC formation. The latter can be interpreted as a form of epigenetic memory, defined as the memory of cellular experience that influences later transient phenotypes across multiple generations (as illustrated in Figure 10). Our experimental data meets the prerequisites for this as follows: the production of PCs-II across multiple generations under a unified LC condition following a BC or LC experience (Figure 8), the transient existence of the PCs-II (Figure 8E,F and Figure 9B), and the nature of this phenomenon as an intracellular event (Figure 9C). In the experiments shown in Figure 8, we considered the theoretical possibility of carryover of small numbers of PCs-I and their inclusion in the PC-II count. However, even when we subtracted the PC-I data, the trends in all data remained consistent with those without subtraction. Therefore, the presence of residual PCs-I has virtually no impact on the interpretation of the PC-II data.

Regarding possible epigenetic memory in bacteria, several recent studies have reported similar findings [14,22,23,24,25,31]. For example, Lee et al. [22] reported that *Pseudomonas aeruginosa* cells enhance their biofilm-forming ability by remembering the experience of exposure to a solid surface across several generations. Bhattacharyya et al. [25] recently reported that *E. coli* cells remember a prior experience of swarming when they encounter a new surface, thereby improving their future swarming efficiency until the fourth generation. Hossain et al. [32] proposed the existence of “primed cells” that can produce *E. coli* PCs across several generations, although the specific priming factor or condition remains unidentified. Our finding is one of the earliest examples of possible epigenetic memory in PC formation and the first example with regard to PC-II formation directed by prior biofilm experience. Although the mechanisms of epigenetic memory in these examples, including ours, remain largely unclear, Bhattacharyya et al. [25] reported that cells store memory in the form of cellular iron levels, which is termed as “iron memory”, in their system. Several epigenetic mechanisms have also been proposed in eukaryotic and bacterial systems [33,34,35]. Nutrition/energy availability and various stresses are known to be the environmental factors related to the induction and control of PCs [36,37,38]; however, there is no data yet showing a relationship with the memory phenomenon observed in this study. Hypothetically, the memory phenomenon discovered in this study may help maintain a high PC production potential in a certain subpopulation of BC-derived cells, even when they detach from the biofilm and reinitiate growth as planktonic cells elsewhere.

## 5. Conclusions

This study initially demonstrated the utility of the filamentation method for efficient PC detection. Using this method, we discovered multiple dormancy levels of PCs-I and a novel effect of biofilm on PC-II formation; the latter also indicated the presence of a multigenerational memory mechanism involved in PC-II formation. Further studies on this novel effect can provide insights into the unique mechanisms of bacterial epigenetic memory and their physiological, biological, and medical significance in latent inducement of PC formation. In particular, these elucidations will make it possible not only to predict the appearance of PCs but also to prevent their development in advance. Alternatively, if the mechanisms of epigenetic memory are elucidated in detail, they may be applicable to the control of some specific bacterial phenotypes via non-genetic means.

## Data Availability

The original contributions presented in the study are included in the article/Supplementary Material; further inquiries can be directed to the corresponding author.

## Supplementary Materials

The following supporting information can be downloaded at https://www.mdpi.com/article/10.3390/microorganisms12091888/s1: Figure S1—original microscopy image of Figure 2A,C; Figure S2—original microscopy image of Figure 2B,D; Figure S3—original microscopy image of Figure 2E,G; Figure S4—original microscopy image of Figure 2F,H; Figure S5—original microscopy image of Figure 7A,C; Figure S6—original microscopy image of Figure 7B,D; Figure S7—original microscopy image of Figure 7E,G; Figure S8—original microscopy image of Figure 7F,H; File S1—explanations of Figures S1–S9; resuscitation data of deeply dormant PCs-I derived from LC.

## Abbreviations

PCs, persister cells; PCs-I, type I PCs; PCs-II, type II PCs; VBNC, viable but nonculturable; BC, air–solid biofilm culture; LC, liquid culture; colony method, conventional colony-forming method; filamentation method, modified cell filamentation method; Cep, cephalexin; PI, propidium iodide; CTC, cyanoditolyl tetrazolium chloride; LB, Luria–Bertani; PBS, phosphate-buffered saline; CFU, colony-forming unit; ANOVA, analysis of variance.
